# How is equity approached in universal health coverage? An analysis of global and country policy documents in Benin and Senegal

**DOI:** 10.1186/s12939-019-1089-9

**Published:** 2019-12-17

**Authors:** Elisabeth Paul, Céline Deville, Oriane Bodson, N’koué Emmanuel Sambiéni, Ibrahima Thiam, Marc Bourgeois, Valéry Ridde, Fabienne Fecher

**Affiliations:** 1 Université de Liège, Faculty of Social Sciences, Place des Orateurs 3, 4000 Liège, Belgium; 2 Université libre de Bruxelles, School of Public Health, Campus Erasme, Route de Lennik 808, 1070 Brussels, CP 591 Belgium; 3 Université de Parakou, Faculté des lettres, arts et sciences humaines (FLASH) et Laboratoire de recherches sur les dynamiques sociales et le développement local (Lasdel), Parakou, Benin; 4 University of Thiès, Research Center in Economics and Applied Finance (CREFAT), Thiès, Senegal; 5 Université de Liège, Faculty of Law, Political Science and Criminology, and Tax Institute, Place des Orateurs 3, 4000 Liège, Belgium; 6 IRD (French Institute for Research on Sustainable Development), CEPED (IRD-Université de Paris), Universités de Paris, ERL INSERM SAGESUD, 45 rue des Saints Pères, 75006 Paris, France

**Keywords:** Universal health coverage, Equity, Global reports, Policy documents, Low- and middle-income countries, Benin, Senegal

## Abstract

**Background:**

Equity seems inherent to the pursuance of universal health coverage (UHC), but it is not a natural consequence of it. We explore how the multidimensional concept of equity has been approached in key global UHC policy documents, as well as in country-level UHC policies.

**Methods:**

We analysed a purposeful sample of UHC reports and policy documents both at global level and in two Western African countries (Benin and Senegal). We manually searched each document for its use and discussion of equity and related terms. The content was summarised and thematically analysed, in order to comprehend how these concepts were understood in the documents. We distinguished between the level at which inequity takes place and the origin or types of inequities.

**Results:**

Most of the documents analysed do not define equity in the first place, and speak about “health inequities” in the broad sense, without mentioning the dimension or type of inequity considered. Some dimensions of equity are ambiguous – especially coverage and financing. Many documents assimilate equity to an overall objective or guiding principle closely associated to UHC. The concept of equity is also often linked to other concepts and values (social justice, inclusion, solidarity, human rights – but also to efficiency and sustainability). Regarding the levels of equity most often considered, access (availability, coverage, provision) is the most often quoted dimension, followed by financial protection. Regarding the types of equity considered, those most referred to are socio-economic, geographic, and gender-based disparities. In Benin and Senegal, geographic inequities are mostly pinpointed by UHC policy documents, but concrete interventions mostly target the poor. Overall, the UHC policy of both countries are quite similar in terms of their approach to equity.

**Conclusions:**

While equity is widely referred to in global and country-specific UHC policy documents, its multiple dimensions results in a rather rhetorical utilisation of the concept. Whereas equity covers various levels and types, many global UHC documents fail to define it properly and to comprehend the breadth of the concept. Consequently, perhaps, country-specific policy documents also use equity as a rhetoric principle, without sufficient consideration for concrete ways for implementation.

## Background

Universal health coverage (UHC) means that all people receive the health services they need, including health initiatives designed to promote better health, prevent illness, and to provide the treatment, rehabilitation and palliative care of sufficient quality to be effective while at the same time ensuring that the use of these services does not expose the user to financial hardship [1]. Equity seems inherent to the pursuance of UHC: for instance, the World Health Report 2008 defines universal coverage reforms as “reforms that ensure that health systems contribute to health equity, social justice and the end of exclusion, primarily by moving towards universal access and social health protection” [2]; the World Health Organisation (WHO) believes that equity is an intermediate objective of UHC [3]; and the WHO Consultative Group on Equity and Universal Health Coverage urges countries to commit to fairness, equity and rights to health in policymaking [4]. However, equity is not a natural consequence of the implementation of UHC policies. On the contrary, the pursuance of UHC implies trade-offs which are not necessarily favourable to vulnerable people, and some policies pursued in the name of UHC may worsen inequalities [5–8]. Hence the acknowledged importance of measuring inequity, and tracking progress in this regard when implementing UHC policies [9, 10].

Note first that equity is a commonly used term in public health. A narrative review of peer-reviewed literature published in English between 2005 and 2013 retrieved approximately 9000 papers from PubMed via the search words, ‘universal health coverage/care’ and ‘equity/inequity’ [9]. However, it is a controversial, ambiguous concept that is intertwined with a number of other concepts such as fairness (which is a broader concept and specifically focuses on the worst-off), equality, social justice, social inclusion, solidarity, altruism, and rights to health [4, 6, 11–15]. It is opposed to health inequalities or disparities, which refer to health differences that are avoidable, unnecessary, and unjust [16]. Health inequities are also closely interconnected with disparities in social determinants of health [17].

Equity in health encompasses various dimensions, some related to means or processes, some related to ends or outcomes [13]: equity in healthcare coverage (access, use of services) (often called *horizontal equity*: equal treatment for equal need); equity in health outcomes; equity in health financing (often called *vertical equity*, meaning that everyone contributes to health financing according to one’s ability to pay); equity in financial protection. When associated to the pursuance of UHC, studies from low- and middle-income countries generally explore the equity impact of UHC based on disaggregated data by geographical area, socio-economic status and gender; but another key area in which inequity may arise across both developing and developed contexts is disparities in the quality of care and access to specialised clinical services [9]. Other types of disparities in health services relate to race/ethnicity, culture, education, or other social advantages [17–21]. The measurement of health inequalities remains challenging and is an evolving concept [9, 12, 22–25].

## Methods

This paper aims to explore on the one hand how the multidimensional concept of equity has been approached in key global UHC reports and policy documents, and on the other hand whether and how this understanding impacts on UHC policies at country level. To do so, we analysed a sample of key UHC reports and policy document both at the global level and in two countries: Benin and Senegal. These two countries are the focus of our research project (2015–2019) on UHC policies. They are located in Francophone Western Africa, have relatively similar health systems and challenges, but have chosen very different paths to expand financial protection coverage for healthcare: while Senegal has opted for community-based health insurance (CBHI), Benin is striving to develop a national, state-led health insurance [26]. This offers interesting comparisons.

We used a similar heuristic approach to the one used by other authors regarding quality in health systems policy [27], and searched the websites of the two major global institutions shaping UHC policies and in charge of its global monitoring – the WHO and the World Bank (WB) – for reports and policy documents dedicated to UHC. We selected the most relevant and significant ones to build a purposeful sample of 20 key UHC reports and policy documents issued after the release of the first World Health Assembly Resolution on UHC (58.33) in 2005 (actually starting in 2008) and until 2018. Note that we excluded two documents whose primary focus was to comprehend equity – the report of the Commission on social determinants of health [17], and the WB’s database of equity indicators [28] – but we also identified and included a report of the International Labour Organisation (ILO) dedicated to social protection in health [29]. We manually searched each document for its use and discussion of “equit*”, “inequal*”, “unequal”, and “disparit*”. The content was then summarised and analysed, especially in order to understand how these concepts were comprehended in each document and to appraise the extent to which the documents were preoccupied by those concepts. This was done through a mixed approach comprising a qualitative content analysis component (thematic analysis of the discourse utilised to approach equity in the documents) and a quantitative component (counting the number of occurrences of the terms related to equity in each document, as well as the breadth of its understanding – levels and types of equity referred to). We distinguished between (i) the *level* at which inequity takes place (social determinants, health outcomes, health systems and policies, access/coverage/use of health services, funding, and financial protection), and (ii) the origin or *types* of health inequities (socio-economic, gender-based, etc.). Then, we adopted the same approach to analyse how the concept of equity was comprehended in three types of UHC policy documents in Benin and Senegal: (i) the national health sector plan (NHSP), (ii) the national health financing strategies (NHFS) for UHC; and (iii) other policy documents describing the UHC strategy or scheme favoured by the government. The results are presented in summary tables and the most salient features are then explained more into details.

## Results

### Equity in key global UHC reports and policy documents

Table 1 offers a quantitative overview of the importance given to equity, and of the dimensions comprehended, in key global UHC policy documents.

**Table 1:** Summary analysis of global UHC policy documents

Document No.	Source	Title	# pages	# occurrences “equit*”	# occurrences “equal*”	# occurrences “disparit*”	Levels of equity considered							Types of inequity considered (stratifiers)							
							Broadly speaking/ undefined	Social determinants and/or health behaviours and/or risk factors	Health outcomes	Health systems and/or policies and/or distribution of resources/funds/expenditure	Access to (quality) healthcare/services and/or coverage and/or use of services	Funding/ financing (contribution)	Financial protection (access) and/or benefit entitlement and/or risk equalisation	Across countries	Income/ wealth/ poverty	Education	Undefined / other socio-economic aspects (e.g. occupation, financial protection schemes)	Geographic: regions and/or urban/rural	Gender	Age	Culture/ ethnicity/ religion/ migrants
G1	WHO 2008	World Health Report 2008: Primary health care: Now more than ever [2]	148	168	82	5	**x**	**x**	**x**	**x**	**x**	**x**		**x**	**x**	**x**	**x**	**x**	**x**	**x**	**x**
G2	WHO 2010	World Health Report 2010: Health Systems Financing: The path to universal coverage [30]	128	41	32	1	**x**	**x**	**x**	**x**	**x**	**x**	**x**		**x**		**x**	**x**			**x**
G3	WHO 2013	World Health Report 2013: Research for Universal Health Coverage [31]	168	27	1	0	**x**			**x**	**x**		**x**		**x**						
G4	WHO 2013	Arguing for Universal Health Coverage [32]	40	23	2	0	**x**					**x**	**x**		**x**		**x**	**x**	**x**		**x**
G5	WHO 2013	Universal Health Coverage: Supporting Country Needs [33]	12	9	7	0	**x**	**x**			**x**		**x**		**x**	**x**		**x**	**x**	**x**	**x**
G6	WHO & WB 2013	Background document Towards UHC: concepts, lessons and public policy challenges [34]	4	2	3	0					**x**	**x**			**x**		**x**				
G7	WB 2013	The Impact of Universal Coverage Schemes in the Developing World [35]	151	21	9	0	**x**		**x**	**x**	**x**										
G8	Rockefeller Foundation, Save the Children, UNICEF and WHO 2013	Universal Health Coverage: A Commitment to Close the Gap [36]	84	373	26	4	**x**	**x**	**x**	**x**	**x**	**x**	**x**	**x**	**x**	**x**	**x**	**x**	**x**	**x**	**x**
G9	WHO & WB 2014	Monitoring progress towards universal health coverage at country and global levels – Framework, measures and targets [25]	14	20	0	0					**x**		**x**		**x**			**x**	**x**		
G10	ILO 2014	Universal Health Protection: Progress to date and the way forward [29]	130	52	20	0		**x**		**x**	**x**	**x**		**x**	**x**		**x**	**x**	**x**	**x**	**x**
G11	WB 2013	Going Universal – How 24 Developing Countries Are Implementing Universal Health Coverage Reforms from the Bottom Up [37]	289	51	27	0			**x**	**x**	**x**	**x**	**x**		**x**		**x**	**x**	**x**		
G12	WHO/ regional office for Africa 2015	The African Health Monitor Special issue: Universal Health Coverage [38]	76	51	6	5				**x**	**x**	**x**	**x**		**x**			**x**			
G13	WHO & WB 2015	Tracking universal health coverage: first global monitoring report [1]	98	35	22	11	**x**	**x**	**x**	**x**	**x**		**x**	**x**	**x**	**x**	**x**	**x**	**x**		
G14	WHO 2016	Health financing country diagnostic: a foundation for national strategy development [39]	58	62	22	0				**x**	**x**	**x**	**x**		**x**		**x**	**x**			
G15	WHO 2016	Public Financing for Health in Africa: from Abuja to the SDGs [40]	92	12	1	0				**x**	**x**		**x**				**x**				
G16	WHO 2017	Global Report: New Perspectives on Global Health Spending for Universal Health Coverage [41]	40	4	2	0	**x**					**x**		**x**			**x**				
G17	WHO 2017	Together on the road to universal health coverage – a call to action [42]	38	9	10	0	**x**			**x**	**x**				**x**		**x**		**x**	**x**	
G18	WHO 2017	Developing a national health financing strategy: a reference guide [43]	44	20	2	0				**x** [UHC intermediate obj.]	**x** [final coverage goal]	**x**	**x** [final coverage goal]				**x**	**x**			
G19	WHO & WB 2017	Tracking universal health coverage: 2017 global monitoring report [4﻿3﻿]	88	12	43	0	**x**				**x**		**x**	**x**	**x**	**x**		**x**	**x**	**x**	
G20	WHO/Regional Office for Africa 2017	The state of health in the WHO African Region: an analysis of the status of health, health services and health systems in the context of the SDGs [4﻿4﻿]	184	38	(8)*	2	**x**	**x**	**x**	**x**	**x**	**x**	**x**	**x**							
**Total (out of 20 documents):**							**12**	**7**	**7**	**13**	**18**	**12**	**14**	**7**	**15**	**5**	**12**	**13**	**10**	**4**	**6**

Note: The number of occurrences presented is the number of *relevant* occurrences: excluding in references and index/contents, and excluding adverbial use of equal* [equally, equal to, etc.]

* SDGs

Regarding the *levels* of equity most often considered, access (availability, coverage, provision, use) is the most often quoted dimension (explicitly referred to by 18 documents out of 20), followed by equity in financial protection (14/20 documents), and then equity at the level of health systems, policies and/or distribution of resources (13/20 documents), with varying focus according to sources. Other dimensions include equity in financial contribution (funding), in health outcomes, and in social determinants of health (incl. distribution of power, money and resources; health behaviours; water and sanitation). Regarding the *types* of equity considered, those most referred to are socio-economic (wealth/income disparities) (explicitly referred to by 15 documents out of 20), geographic (across regions and urban/rural areas) (mentioned by 13/20 documents), other or undefined socio-economic disparities (12/20 documents), and gender-based disparities (10/20 documents). A variety of other criteria (“stratifiers”) are mentioned throughout the documents: education, age, and cultural factors such as religion, race/ethnicity, and migrant status.

A more in-depth and qualitative content analysis of how equity has been approached in global UHC documents is provided in Table 2.

**Table 2:** Analysis of the way equity is approached in global UHC documents

Document no.	Source	Title	Explicit definition of equity?	How equity is approached in the document
C1	WHO 2008	World Health Report 2008: Primary health care: Now more than ever [2]	No	- Chapter 1 “The challenges of a changing world” devotes a section to the “Changing values and rising expectations” which comprises a subsection on “Health equity” - Equity is a **central concern** of Chapter 2 “Advancing and sustaining universal coverage”, especially in the following sections: “The central place of health equity in primary health care (PHC)” and “Mobilizing for health equity” - Among the four reforms advocated for in the report, universal coverage reforms are viewed as those that “ensure that health systems contribute to health **equity, social justice and the end of exclusion**, primarily by moving towards universal access and social health protection” (page ix, message of the Director General -- bold ours) - The report points to the **multiple dimensions** of health inequality - Associations of equity with UHC and other concepts: o The report explains the **links between UHC and equity** – mostly through social health protection, increasing financial access to healthcare (by contrast, out-of-pocket payments (OOPs) are denounced as inequitable) o Equity is viewed as a **value driving the primary healthcare movement**, together with **solidarity** and **social justice**
C2	WHO 2010	World Health Report 2010: Health Systems Financing: The path to universal coverage [30]	No	- The report argues in chapter 3 that compulsory prepaid funds, if possible pooled into a single pool, enables to achieve equity goals – sometimes called equity funds - It makes the case for decisions that contribute to equity in contributions, in pooling, and in use of resources - Associations of equity with UHC and other concepts: o Equity is associated repeatedly with **efficiency** o It is mentioned once with **fairness** and **basic decency**
C3	WHO 2013	World Health Report 2013: Research for Universal Health Coverage [31]	No	- The report has a section dedicated to “**Equity and universal health coverage**” - It points to the “inequitable access to the products of research” (p. 45) - Associations of equity with UHC and other concepts: o Equity is associated with **cultural values, right to health and social justice** as well as with discrimination o It is also associated with **quantity and quality** of services, and with **efficiency**
C4	WHO 2013	Arguing for Universal Health Coverage [32]	Yes: “Equitable: does the mechanism raise funds according to people’s ability to pay and are the benefits distributed according to people’s health needs?” (p. 25)	- The document focuses on the **equity of the health financing system**, and provides the case for “health funding policies that promote equity, efficiency and effectiveness, and ensure that the rights of the most vulnerable are not forgotten” - It singles out out-of-pocket financing as failing badly in terms of equity and financial risk protection - Associations of equity with UHC and other concepts: o Equity is associated with **efficiency and effectiveness**, and (human) **rights** (to health) o The document considers that **equity is part of UHC**: “equity and financial risk protection, which are integral to achieving progress towards UHC “(p. 25); “the equity criterion fundamental to attaining UHC” (p. 27); “the equity principles that should be the foundation of any UHC strategy” (p. 32)
C5	WHO 2013	Universal Health Coverage: Supporting Country Needs [33]	Yes: “Equity: If all people obtain the health services they need without suffering financial hardship, equity in access has been achieved” (p. 9)	- Associations of equity with UHC and other concepts: The document considers **equity is part of UHC**: “This vision of UHC embodies principles of equity in access to and use of services, quality of the services people obtain, and financial protection for people needing health services” (p. 5); UHC is not only about health but also “moving closer to UHC is also about equity, development priorities, social inclusion and cohesion” (p. 10); **UHC “is a concept that is fundamentally about equity**” (p. 12)
C6	WHO & WB 2013	Background document Towards UHC: concepts, lessons and public policy challenges [34]	No	- The document makes the **case for health financing reforms**: “the objectives of universal financial protection and equity in the use of needed services are best served when health systems rely predominantly on compulsory prepaid funds” - It also makes the case for **strengthening the primary level of care**
C7	WB 2013	The Impact of Universal Coverage Schemes in the Developing World [35]	No	- Associations of equity with UHC and other concepts: o **UHC** viewed as a **means to increase equity** o Equity associated with **efficiency (and quality**)
C8	Rockefeller Foundation, Save the Children, UNICEF and WHO 2013	Universal Health Coverage: A Commitment to Close the Gap [36]	Yes: “inequity – unfair and avoidable inequalities” (p. 4 and later)	- Equity is a **central concern** of this report: “This report focuses on how and why inequity – unfair and avoidable inequalities – should be prioritised as countries progress on the path towards UHC” (p. 4): o The report refers to all types and levels of equity identified in Table 1 o Section 2 explains why equity is important for UHC o Section 3 provides a conceptual framework for assessing equity in pathways to UHC, and then lessons for equitable pathways towards UHC o Section 4 makes the economic case for equitable pathways towards UHC - The report specifies that “Within health systems, equity applies to the goals of improved health outcomes, equity in finance, financial risk protection and responsiveness, as well as the objectives of good quality and utilisation based on need” (p. 15) - Associations of equity with UHC and other concepts: o **UHC** viewed as a **means to achieve greater equity**, or “the response to” inequities (p. 6); and as **necessitating consideration for equity throughout reform processes** o Equity related to **fairness**; calls to “a **moral and ethical** perspective” (p. 4)
C9	WHO & WB 2014	Monitoring progress towards universal health coverage at country and global levels – Framework, measures and targets [25]	No, but definition of equity indicators of coverage and financial protection	- The framework for monitoring progress towards UHC monitoring of UHC puts a **focus on equity** regarding the two discrete components of health system performance (coverage of health services and financial protection), and recommends that “Measures should be disaggregated by socioeconomic and demographic strata” (p. 5) - The global framework proposes **three primary elements for disaggregation** that can be measured comparably in all settings: household income, expenditure or wealth (coverage of the poorest segment of the population as compared with richer segments); place of residence (rural or urban); and gender (p. 6) - Each country is expected to add further measures of service coverage and further equity stratifiers in order to tailor UHC monitoring to its context (p. 10) - Recommended indicators comprise **an aggregate and an equity measures** - Associations of equity with UHC and other concepts: o Reckons that “At the heart of **UHC** is a **commitment to equity**” (p. 6)
C10	ILO 2014	Universal Health Protection: Progress to date and the way forward [29]	No	- Mentions that OOPs are the most inequitable source of health financing (p. 2); their removal can help progress in terms of “effective and equitable access to health care, affordability and financial protection in addition to availability of quality services” (p. 6) - Refers to “inequities in legal health coverage due to political, legislative and administrative failures” (p. 2) - Has a section on “**Moving towards Equity**: National Social Protection Floors as a key strategy for achieving universal coverage in health” - Associations of equity with UHC and other concepts: o **UHC and equity** viewed as **two distinct aims** (p. iii) o Equity associated with **human rights** to social security and health and the **rights-based approaches** underpinning the need for equity and poverty alleviation (p. iii); with **social change**, **poverty alleviation** the **elimination of deprivation** (pp. 4, 47); with **social justice** (pp. 9, 77); with **vulnerability and social exclusion** (p. 37); with **universality** (p. 39); with **solidarity** (p. 66); with **social fairness** (p. 72); with **inclusion** (p. 111) o Mentions the **trade-off between equity and quality** of essential health services (p. 45)
C11	WB 2015	Going Universal – How 24 Developing Countries Are Implementing Universal Health Coverage Reforms from the Bottom Up [37]	No	- Points repeatedly to the **trade-off between equity** in the benefit package **and (fiscal) sustainability** - Associations of equity with UHC and other concepts: o Equity repeatedly associated with “better results for the money spent” (p. xiv) / with **efficiency** and **effectiveness** o Also associated with **sustainability, accessibility, quality, integration; implementability**
C12	WHO/ regional office for Africa 2015	The African Health Monitor Special issue: Universal Health Coverage [38]	Not in general, but vertical equity is defined as “cross-subsidization from wealthy to poor” (p. 24)	- Several chapters are focused on the equity aspects of community-based health insurance - A case study in Senegal focuses on **vertical equity** – and mentions that it “is likely to overlap with the “health risk” dimension of **solidarity**” (p. 24) - Another case study in Senegal reckons that the **equity paradigm has been developed at the international level**, as a consequence of financial barriers that have reduced utilisation of healthcare by the poorest (p. 63) - Associations of equity with UHC and other concepts: o Equity repeatedly associated with **efficiency** o Also associates equity with **adequacy** (of coverage) and **sustainability** (p. 51); with **universality** and **solidarity** (p. 59–60) o The Regional Director mentioned the “shared values of equity, **dignity, transparency, integrity, professionalism and openness**”; inequities also compared to **injustices** (p. 67)
C13	WHO & WB 2015	Tracking universal health coverage: first global monitoring report [1]	No	- The first global monitoring report of UHC regret that “Because of the **lack of data**, it is not yet possible to compare the UHC service coverage index across key dimensions of inequality” (p. viii) - A key challenge is to monitor equity in access to quality health services (p. 4) - Associations of equity with UHC and other concepts: o The report states that “Equity is key to the **SDGs** in general and to **UHC** specifically” (p. xii) – and recalls **SDG 3**: Equitable health outcomes and well-being; global public health security and resilient societies (p. xiii)
C14	WHO 2016	Health financing country diagnostic: a foundation for national strategy development [39]	Yes, partly: makes the distinction between different utilisations of the concept of equity (see next column)	- Specifies that “equity in the use of services refers to reducing the gap that exists between the need for a health service and the actual use of that service” (p. 3) - Defines **equity in finance**, which “is strongly related to the goal of financial protection, but is conceptually distinct. Equity in finance refers to the distribution of the burden of financing the health system across different socio-economic groups. To be considered equitable, the burden of health financing should be distributed according to individuals’ ability-to-pay” (p. 3) - It is distinct from **equity in financing** which “has to do with how revenues are raised, not with how the money is spent” (p. 23) - The report has a subsection on “Financial protection and equity in finance” (pp. 21–25) and another one on “Equity in service use and in the distribution of resources” (pp. 26–27) - It makes a distinction between equity in health finance and equity in financing: “Equity in financing has to do with how revenues are raised, not with how the money is spent . This latter issue – also highly relevant to the performance of health financing arrangements – is addressed below in the section on equity in health service use and the distribution of system resources.” - Box B2 shows a summary of key findings from previous studies on equity in financing (pp. 44–45) - Associations of equity with UHC and other concepts: o Equity is approached through the links between health financing, UHC goals and intermediate objectives – indeed, **equity in service use is a UHC goal**, and the **distribution of resources is a UHC intermediate objective** o Thus association of equity with other UHC objectives/goals: **efficiency, transparency and accountability, quality, financial protection**
C15	WHO 2016	Public Financing for Health in Africa: from Abuja to the SDGs [40]	No	- Associations of equity with UHC and other concepts: o Equity is associated with **sustainability** (pp. 8, 33), **quality** (p. 22), **efficiency** (pp. 30, 33)
C16	WHO 2017	Global Report: New Perspectives on Global Health Spending for Universal Health Coverage [41]	No	- Associations of equity with UHC and other concepts: o Equity is associated with **social cohesion** (p. 4), **sustainability** (p. 8) o Intends to promote “equitable progress towards UHC” (p. 29), thus differentiating the two concepts
C17	WHO 2017	Together on the road to universal health coverage – a call to action [42]	No	- Associations of equity with UHC and other concepts: o Equity is associated with **efficiency** (p. 14), **human rights** (pp. 18, 20), **gender equality** (p. 20)
C18	WHO 2017	Developing a national health financing strategy: a reference guide [43]	No	- Associations of equity with UHC and other concepts: o Considers *equity in utilisation or service use* relative to need as part of a **normative set of goals embedded in the concept of UHC**, together with **financial protection** and **quality**; *equity in the distribution of health system resources* as part of a **set of intermediate objectives,** together with **efficiency**, **transparency and accountability** (p. 1) o Equity also associated with **effectiveness** and the management of expenditure growth (p. 12)
C19	WHO & WB 2017	Tracking universal health coverage: 2017 global monitoring report [4﻿3﻿]	No	- Has a lot in common with the first global monitoring report on UHC as for its approach of equity (lack of data preventing comparing the UHC service coverage index across key dimensions of inequality, link with SDGs and UHC, etc.) - Associations of equity with UHC and other concepts: o The report reckons that “Unless health interventions are designed to promote equity, efforts to attain UHC may lead to improvements in the national average of service coverage while inequalities worsen at the same time” (p. viii) – therefore making a **clear distinction between UHC and equity**
C20	WHO/Regional Office for Africa 2017	The state of health in the WHO African Region: an analysis of the status of health, health services and health systems in the context of the SDGs [44]	No	- The report highlights the inequities between the countries of the WHO African region, and also within countries - It originally mentions the inequities in the countries health security status (p. 33) - It notices the interconnection between the different levels of inequity: “These inequities in health are a result of inequities in investments in and outcomes from these investment” (p. 83) - Associations of equity with UHC and other concepts: o Equity is associated repeatedly with **sustainability**, **efficiency and effectiveness;** and once with **resource adequacy** (p. 71) and **human rights** (p. 84) o The report notices that the 2030 Agenda for **Sustainable Development** has “a strong focus on equity” (p. 1) o It also states that “progress towards UHC and the SDGs, particularly from the equity perspective” (p. 83), suggesting that equity is a dimension of UHC & SDGs

Analysis of that sample of key UHC policy documents offer a number of interesting comments. First, most documents (15/20) do not define equity in the first place. Second, most documents speak about “health equity” or “inequity in health” in the broad sense, without mentioning the dimension or type of inequity considered. Third, there is a certain ambiguity in some dimensions of equity: “coverage” is sometimes utilised in the sense of access or availability of health services, sometimes in the sense of utilisation of health services; and “financing” sometimes refers to equity in funding (mobilisation of resources, vertical equity, public spending), sometimes to equity in resource allocation, and even sometimes in financial protection. Fourth, most documents assimilate equity to an overall objective or guiding principle closely associated to UHC and the Sustainable Development Goals (SDGs). However, the links alluded to between UHC and equity are not straightforward: overall, it is unclear whether a focus on equity is supposed to enable progress in UHC (equity as a means), whether UHC is supposed to increase equity (equity as an end), or whether these are two distinct aims. Similarly, it is unclear whether equity is a value or principle orienting actions, or whether it is an objective of such actions. Some of the documents try and clarify the links between UHC and equity: for instance, the publications from the health financing department of the WHO recurrently use a model stating that equity in the distribution of resources is a UHC intermediate objective, and equity in service use is a UHC goal [39, 45]; a joint report views UHC as “the response to” inequities [36]; and the 2017 Global monitoring report makes a clear distinction between equity and UHC [4﻿3﻿]. Nevertheless, the global picture is unclear in most documents, and the approach of equity is often more rhetorical than concrete. Fifth, the concept of equity is also often linked to other concepts and values such as social justice (or inclusion), solidarity, human rights (including the right to health), and poverty alleviation – but it is even more often associated with efficiency, as well as with sustainability.

### Implications on country UHC policies

Table 3 and Table 4 follow a similar thematic analytical approach and summarise the results from the analysis on how the concept of equity was comprehended in three types of country-specific policy documents in Benin and Senegal: (i) the national health sector plan (NHSP), (ii) the national health financing strategy (NHFS); and (iii) other UHC policy documents.

**Table 3:** Summary analysis of UHC policy documents in Benin and Senegal

Document no.	Source	Title	# pages	# occurrences “equit*”	# occurrences “equal*”	# occurrences “disparit*”	Levels of equity considered							Types of inequity considered (stratifiers)						
							Broadly speaking/ undefined	Social determinants and/or health behaviours and/or risk factors	Health outcomes	Health systems and/or policies and/or distribution of resources/funds/expenditure	Access to (quality) healthcare/services and/or coverage and/or use of services	Funding/financing (contribution)	Financial protection (access) and/or benefit entitlement and/or risk equalisation	Income/ wealth/ poverty	Education	Undefined / other socio-economic aspects (formal/informal, financial protection schemes)	Geographic: regions and/or urban/rural	Gender	Age	Culture/ ethnicity/ religion/ migrants
C1	Senegal/MoH 2009	PNDS (NHSP) 2009–2018 [46]	86	13	5	5	x	x		x	x						x			
C2	Senegal/MoH 2013	Strategic plan for the development of universal health insurance in Senegal 2013–2017 [47]	127	13	2	0					x	x				x				
C3	Senegal/MoH 2017	Sector Investment Plan 2017–2021 [48]	25	2	4	0				x	x		x			x				
C4	Senegal/MoH	Strategic development plan of the Agency for universal health insurance [49]	66	5	21	6					x	x	x			x				
C5	Senegal/MoH 2017	National health financing strategy (NHFS) [50]	33	16	7	6				x	x	x	x	x			x	x		
C6	Benin/MoH 2010	PNDS (NHSP) 2009–2018 [51]	96	6	5	7			x	x	x	x		x		x	x	x		
C7	Benin/MoH 2015	National health financing strategy (NHFS) 2016–2020 [52]	43	21	1	1			x	x	x	x	x			x	x			
C8	Benin / Government 2019	Project document: Insurance for the strengthening of human capital (ARCH) [53]	45	9	4	2		x			x			x		x	x			
**Total (out of 8 documents):**							**1**	**2**	**2**	**5**	**8**	**5**	**4**	**3**	**0**	**6**	**5**	**2**	**0**	**0**

Note: The number of occurrences presented is the number of *relevant* occurrences: excluding in references and index/contents, and excluding adverbial use of equal* [equally, equal to, etc.]

**Table 4:** Analysis of the way equity is approached in UHC policy documents in Benin and Senegal

Document no.	Source	Title	Definition of equity?	Way equity is approached in the document
C1	Senegal/MoH 2009	PNDS (NHSP) 2009–2018 [46]	No	- The Plan states that more than before, **equity in health service distribution and financial access** (financing the demand-side) were **prioritised** - Strategies announced to improve equity in health service distribution: o Making a **minimum healthcare supply capacity available in the regions** (including creating district hospitals) o **Revising the health map** (norms in terms of infrastructure, equipment and personnel per level of care) to make it more ambitious in terms of supply capacities of services / ensuring a better distribution of health facilities throughout the country o Improving the resource allocation system (not further explained) - A number of measures are announced to reinforce the regulatory function of the State, including regarding issues of **equity, gender, discrimination, and social protection** - Associations of equity with UHC and other concepts: o Equity is viewed as a **principle guiding the implementation of the NHSP**, together with participation, multisectoriality, transparency, solidarity, and gender o Inequities are also associated with exclusion
C2	Senegal/MoH 2013	Strategic plan for the development of universal health insurance in Senegal 2013–2017 [47]	No	- The strategic plan starts from the observation that the evolution of the country’s health system has not promoted equity in access to health care, household financial protection, and equity in health financing; however, free and subsidised healthcare initiatives have enabled to increase equity - The plan refers to the 2012 UNGA resolution on UHC which calls on each UN Member State to avoid out-of-pocket payments and to finance its health system through more equitable and supportive mechanisms - The plan intends to **reform the health financing system by expanding health insurance to rural and informal sectors, through promotion of community-based health insurance (CBHI)** and subsidisation of premiums for the poorest, and the creation of the National Health Solidarity Fund - Progressiveness in health financing is to be ensured through the development of information systems to scale **contributions based on households’ ability to pay for health care** (not further developed) - The logical framework of the plan has an impact indicator of equity of access, measured through health service utilisation rates - Associations of equity with UHC and other concepts: o The values and principles of the plan are: **solidarity, equity and social justice, quality, efficiency, good governance, and partnership** o Equity is viewed as a **basic principle** to ensure social inclusion, the inclusion of the poor and vulnerable groups, and equitable access to care
C3	Senegal/MoH 2017	Sector Investment Plan 2017–2021 [48]	No	- In order to respond to unequal distribution of infrastructures throughout the country, the plan announces that its priorities were defined taking into account the gaps identified by the “**health and social map**” and **equity criteria** with a focus on high-impact interventions (without further detail) - It will give priority to two essential components: **demand financing** and **construction of new infrastructure**
C4	Senegal/MoH 2017	Strategic development plan of the Agency for universal health insurance [49]	No	- The plan states that respect for equity is a fundamental element in improving access to care and reducing poverty; and it also refers to the 2012 UNGA Resolution on UHC - The plan recalls that the universal health insurance (“CMU”) policy is strongly affirmed as the strategy to ensure equitable access to quality health care without any form of exclusion for the entire population of Senegal, and that it is based on the development of mutual health insurance and the strengthening of free healthcare initiatives - One of the main strategies envisioned is support to the **affiliation of the poorest** (beneficiaries of social assistance programmes, including equal opportunity card holders) **to CBHIs** - Associations of equity with UHC and other concepts: o Equity is part of the Agency’s vision in order to fight exclusion, and of its values under the form of **fair and equal treatment** for every Senegalese
C5	Senegal/MoH 2017	National health financing strategy (NHFS) [50]	No	- The strategy is based on a situation analysis that emphasises the **inequitable distribution of resources**, including human resources, especially between regions and living environments but also between levels of care - In particular, the identified **obstacles to equitable access** to health services are: remoteness, isolation, inadequate accommodation for persons with disabilities, high cost of health services, and lack of availability of health facilities - The compulsory contributory scheme to health insurance for employees in the private sector is also judged inequitable - The strategy reckons that the principle of equity will be clearly positioned in the criteria that support decisions on the **allocation of resources** in order to democratize access to health services - The strategy comprises four strategic orientations: o The first one intends to improve the availability of quality health services, with a **focus on ensuring equitable access** to quality health services – notably through the “densification and democratisation of the supply of health services” (revising the health and social map) o The second one intends to expand protection against health-related financial risk, also with a **focus on equity** through promotion of CBHIs to the rural and informal sectors, subsidisation of the contribution of the poorest, and unification of governance mechanisms of the various UHC schemes o The third one intends to target behavioural and environmental determinants of health o The fourth one intends to raise more resources, and improve their efficiency, notably through the application of budget allocation criteria (not further explained) - Associations of equity with UHC and other concepts: o **Sustainable development** necessitates the respect of the principles of **equity and gender equality** o The strategy is based on a **vision** of a Senegal where all populations have access to quality health services based on sustainable financing that respects the principles of equity and solidarity; the search for equity will be combined with the principle of solidarity to ensure the social inclusion of the poor and vulnerable groups in health risk coverage mechanisms
C6	Benin/MoH 2010	PNDS (NHSP) 2009–2018 [51]	No	- The plan is based on a situation analysis that points to inequitable distribution of staff, as well as financial barriers that do not favour equitable access to healthcare - The plan intends to give priority to **equitable financing** and sound management of health expenditure - Associations of equity with UHC and other concepts: o The **2025 vision** of the plan intends to ensure the permanent availability of quality, **equitable** and accessible healthcare services to populations of all categories, based on the values of solidarity and risk sharing
C7	Benin/MoH 2015	National health financing strategy (NHFS) 2016–2020 [52]	No	- The situation analysis points to **great geographical disparities** – notably in the distribution of human resources – and to the fragmentation of financing schemes that lead to inequitable access to healthcare - The strategy is based on **several principles linked to equity**, including: equitable and easy access to quality health care at affordable costs according to needs, the availability of health care provision to the entire population, solidarity and risk sharing based on obligation and non-exclusion, and protection against financial risk; equitable sources of financing are looked for - The strategy comprises three strategic orientations: o Improve and streamline the **utilisation of resources** in the health sector o Implement the health insurance scheme and integrate other financial protection schemes o Guarantee that the **health financing system** is equitable, sustainable and predictable - Concretely, the proposed measures to improve equity are: o The revision of the Ministry of Health’s **budget allocation procedures** to ensure equity and efficiency; budget should be allocated on the basis of existing resources and performance, in line with and linked to resources from community funding o Populations’ **contribution to the State-led health insurance** scheme in function of their income (and exemption for the poorest) - Associations of equity with UHC and other concepts: o The strategy is based on the same vision as the NHSP 2009–2018; as well as on the following values: (i) accountability and leadership, (ii) equity, social justice, ethics and good governance, and (iii) effectiveness and efficiency o Equity repeatedly associated with **sustainability** and **efficiency**
C8	Benin / Government 2019	Project document: Insurance for the strengthening of human capital (ARCH) [53]	Yes: see next column	- The overall objective of the ARCH project is to increase capacity and access to basic social services and economic opportunities in a sustainable and **equitable** way for Beninese people, especially the poorest - The project comprises **4 packages of social protection services** (health insurance, training, credit and retirement) to improve the impact of programmes and equity in access to basic services - In the context of the implementation of the ARCH health insurance service, equity is approached in two respects: (i) **equity of access**, i.e. facilitating access for all Beninese citizens to the package of basic benefits, according to their individual health needs, and (ii) **contributory (vertical) equity**, which aims to involve each social stratum in the functioning of the ARCH system according to their ability to pay - Associations of equity with UHC and other concepts: o Equity associated with **efficiency, economies of scale** and **sustainability**

The documents from the two countries present similarities, which enables a joint analysis. Note first that a number of findings are similar to those of the global documents. Most country-specific policy documents (7 out of 8) do not define equity in the first place; coverage, access and supply of services are used interchangeably, and the dimensions and types of health equity are often not well explicated. Regarding the level of equity considered, all country documents in our sample explicitly refer to access / coverage / use of services. Country documents also put a lot of emphasis on improving equity in allocation of resources (especially human resources) across regions (referred to explicitly by 5 documents out of 8), and on funding/financial contribution (5/8 as well). Regarding the types of equity considered, undefined or broad socio-economic disparities (e.g. formal vs. informal sector, vulnerable populations) are referred to by 6 documents out of 8, while geographic (including rural/urban) inequalities are the most widely used explicit stratifier. However, in the documents reviewed, no mention was made to inequity in service quality or due to differences in education, age, or cultural aspects. Moreover, the examined country policy documents tend to refer to the principle of equity in a quite rhetoric way, in association with other broad concepts (e.g. solidarity, social justice) but also predominantly with efficiency and sustainability.

The supposedly predominant policy document in each country, that is the *NHSP*, covers the period 2009–2018 in both countries. The NHSP of Benin reckons the existence of inequitable distribution of staff, as well as financial barriers that do not favour equitable access to healthcare, and intends “give priority to equitable financing”. It is based on a vision that intends to “ensure the permanent availability of quality, equitable and accessible healthcare services to populations of all categories, based on the values of solidarity and risk sharing” *(our translation)*. However, no concrete measure to improve equity is proposed in the NHSP [51]. That of Senegal announces that “priority is given to the equitable distribution of the supply of services and the financing of health demand” and that “the provision of a minimum supply of care per region and the judicious spatial distribution of diagnostic and treatment facilities will ensure more equitable care”; this is presumed to be achieved through a “resource allocation system made more equitable” and a “greater attention given to the operationalisation of the health map” *(our translation)*. If more concrete strategies to improve equity are indicated than in the one of Benin, the NHSP of Senegal also refers to equity in a vague, rhetoric way, as a guiding principle among others such as “participation, multisectoriality, transparency, solidarity, and gender” [46]. More concrete actions are to be found in the subsequent Sector Investment Plan that intends to “give priority to two essential components: demand financing and construction of new infrastructure” [48].

Both countries have also issued a *national health financing strategy towards UHC* (NHFS). That of Benin puts the emphasis on equity in financing and considers the need to “find places to collect (taxes, indirect taxes) that meet equity concerns” and to ensure “more equitable distribution of budget allocations” *(our translation)* [52]. That of Senegal diagnoses “an inequitable distribution of (human, material and financial) resources” (especially between regions and living environments, but also between levels of care) and states that “the principle of equity will be clearly positioned in the criteria that support decisions on the allocation of resources in order to democratise access to health services” *(our translation)*. The strategy comprises four strategic orientations, the first two having a focus on equity in access to quality healthcare and financial protection [50]. However, beyond those principled statements, none of these plans proposes any concrete action (such as resource allocation criterion) to advance equity.

Finally, the two countries have chosen different *strategies to increase financial protection coverage*: while Senegal has opted for CBHIs [47, 49], Benin has opted for a State-led project, called ARCH, which comprises for packages of social protection services (health insurance, training, credit and retirement) [53].

Overall, despite differences in contexts and in political choices and discourses, the UHC policies of the two countries are quite similar in terms of their approach to equity. Both countries acknowledge important health disparities – especially geographic ones – and assign them to inequities in resource allocations and on insufficient financial protection. On the supply side, both countries intend to revise budget allocation procedures to ensure equity and efficiency – yet, the documents examined for Benin do not explain how to do so, while the UHC policy documents in Senegal mention the “health map” (i.e., the norms in terms of infrastructure, equipment and personnel per level of care) as a concrete way to do it [46, 48, 50]. On the demand side, both countries intend to increase financial health coverage by promoting a health insurance scheme for the rural and informal sectors, and subsidising the contributions of the poor to those schemes. They also recognise the problems arising from the fragmentation of financing schemes and consider setting up a common pool, but have not yet succeeded in this respect [47, 52]. Finally, both countries are aware of the importance of social determinants of health; Senegal intends to act in this respect through multisectorial action, while Benin has rather opted for developing a whole package of social protection programmes through the ARCH project [50, 53].

## Discussion

Our study has a number of methodological limitations. It is based mostly on a documentary review, and on a purposeful sample of reports and documents. We have deliberately excluded documents targeted on equity so as to appraise whether non-specific UHC documents apprehended this concept. We have based our analysis mostly on the documentary review, without complementary methods other than our personal knowledge and expertise, accumulated during our four-year research project and prior experience in the two focus countries. We have not analysed how these documents were produced, but their origin and formulation process could explain whether or not equity is taken into account.

Our results show that the concept of equity is often used in an imprecise and even rhetoric manner, both in global and in country-specific UHC policy documents. This is true both with respect to the levels of equity considered (access, coverage and use are often used interchangeably) and to the types of equity considered (with often undefined “socio-economic” disparities). Financial equity is particularly misunderstood in many documents under review: most examined documents confuse equity in resource mobilisation and equity in resource allocation – while actually “[e]quity in financing has to do with how revenues are raised, not with how the money is spent” [39].

Our study was based on an analysis of policy documents – but beyond stated policies, there might be important policy-implementation gaps. Our experience in Benin and Senegal shows that the two countries struggle to improve resource allocation and to increase financial protection coverage. In Benin, after the notable failure of the attempt of the previous government to develop a national health insurance scheme, the current government’s ARCH project has endured long delays before it started to be piloted in July 2019 [54]. In Senegal, at the end of December 2018, only 19% of the total population benefitted from health risk coverage through CBHI, against an objective of 26% [55]. According to the recent Global monitoring report, the UHC Service Coverage Index (SDG 3.8.1) was at 39.6 for Benin in 2017 (down from 40.2 in 2015) and at 45.4 for Senegal in 2017 (up from 43.8 in 2015) [56]. How can that be explained? Whereas equity is a central concept in public health (thus as the policy-making level in the health sector), it is not apprehensible as such by all disciplines, which may lead to some difficulties as for translating polices into practice. Indeed, before they can be implemented, policies have to be translated into legal and institutional frameworks. However, the notion of equity merely does not exist as such in law. In Common Law systems, the term “equity” refers to a particular set of doctrines and procedures involved with civil law, which complement the statutory laws, but with no real connection to the meaning used in public health. In civil law legal systems, equity does not exist as a concept, but is comprehended through other concepts such as equality and non-discrimination, protection of minorities, minimum base, proportionality or ability to pay, or fiscal federalism. It can also be addressed through economic and fundamental social rights, including the right to health, which imply positive obligations on behalf of government. Yet, the choice of the legal concept that will be used to translate the equity goal will not be without consequence.

Since equity encompasses many dimensions, a number of questions arise when aiming to increase equity in the context of UHC policies. First, *which aspects of equity should be prioritised*? For instance, should UHC policies guarantee basic rights to the whole population, or target the poorest (or other disadvantaged groups) first? A recent narrative review found that the majority of papers reviewed in public health found that UHC programmes should focus first on increasing coverage and decreasing economic barriers to access amongst the most disadvantaged groups (“progressive universalism”) [9]. However, there is no consensus on how to realise that objective [57]. In particular, the implementation of targeted strategies (compared to universal ones) involve important pitfalls, such as the political unsustainability of the reforms, as well as the fact that “benefits meant exclusively for the poor often end up being poor benefits” [58]. Moreover, the levels and types of equity to be prioritised depend on the values of the society in which it takes place, and should logically vary from one country to another. Yet, we observe that while global documents consider many stratifiers, country-specific documents’ diagnosis focuses on geographic and urban/rural inequities, which are probably the easiest type of disparities to be apprehended with existing data. Paradoxically, in the two countries under study, few concrete interventions are implemented to improve balance in the allocation of resources, while policies targeting the poorest or the most vulnerable exist for instance (e.g. free healthcare for children under five or caesarean sections, subsidisation of the ARCH social health insurance in Benin and adhesion to CBHI in Senegal). There is therefore a mismatch between the diagnosis of problems, and the solutions proposed. The urban bias in political decision in low- and middle-income countries has been known for decades [59]. Domestic political interests may probably explain why the health insurance policy are high in the political agenda of the Presidents of both Benin and Senegal. In Benin, the integrated social protection programme is designed to strengthen the human capital of the country in view of supporting its development, in line with the neoliberal vision of its government [53]. In Senegal, the choice of the CBHI model – against the tide of the international experience pointing to its limited potential to progress towards UHC [60] – was influenced by domestic mutualist lobbies supported by the American cooperation agency [61].

Second, once the equity objective has been defined, the question arises *how to translate it into the country legal and institutional frameworks?* Many alternatives may be possible in this respect, but the similarities in terms of equity objectives between the two studied countries – which have however chosen very different paths to reach UHC – raises questions about the capacity of countries to actually translate global guidelines into practice in a context-specific way. The question of how to translate a moral imperative into practice has been questioned for decades, as testified by the tensions around whether and how to provide “equal opportunity” in society [62]. The development of a UHC policy may be a strictly political agenda, but it may also be pushed back by constitutional or statutory constraints, international agreements or conventions; the UHC policy may have various degrees of binding forces – the right to health may be inscribed up to the level of the constitution (in which case it creates positive obligations for the state to secure the effective enjoyment of it). More generally, social rights and the right to health may have different legal values; if there is a legally binding commitment towards UHC, citizens could claim it directly or indirectly before the authorities, especially the courts. The stand-still rule (meaning that when a certain level of social protection or a right is reached, there is no turning back) may be used to guarantee financial commitments. Beyond access, equity in finance should also be searched for, implying that the distribution of the burden of financing health services is “fair”, thus referring to the extent to which financing is progressive or regressive i.e. whether the burden falls disproportionately on the better-off, or worse-off, in society, relative to their capacity to contribute [63]. As outlined above, the two countries studied intend to render health financing more progressive and to pool resources at a high level, but no major progress has been achieved so far. In Benin, there is still no clear and sustainable mechanisms in place to finance the ARCH project [64]. In Senegal, the legal and institutional frameworks are not totally in line with the UHC policy [65].

Third, once formalised, *how should the implementation of equity policies be facilitated*? Once again, policies could be seductive but difficult to translate into appropriate decrees then implemented, be it for political or technical reasons [57], so wide policy-implementation gaps may take place [66]. Thus the content of policy documents does not necessarily reflect what is done in the field. The two country-specific cases suggest this is likely to happen as they put emphasis on re-equilibrating resource allocation within the health system, without defining allocation criteria or proposing any firm commitment to it – except for the reference to the health map in Senegal. Furthermore, during implementation, UHC policies should be closely monitored since they may have unexpected effects on equity. For instance, a recent study on maternal healthcare in Senegal shows that while a demand-side intervention (abolition of user fees) benefitted the poorer households more, thereby reducing inequity, a supply-side intervention (expansion of the availability of maternal health services) benefitted the richer households more, thereby increasing inequity [67].

Finally, it is worth remarking that improving equity in health necessitates intervention beyond the health sector, and beyond national frontiers. Indeed, UHC is not enough to ensure the right to health, and requires important changes to render the environment healthier [68]. Besides, national policies which target only domestic factors have limited ability to address health inequities, without engaging with the global political economy and acting on global health inequity determinants [69].

## Conclusion

Overall, it appears from our study that while equity is widely referred to in global and country-specific UHC policy documents and seems self-evident, its context-specific interpretations and applications may vary, and the concept is often utilised in a rather rhetoric and/or political way. Whereas the concept of equity covers many levels and types, and except for very specific ones, many global UHC documents fail to define it properly and to comprehend the breadth of the concept. Consequently, perhaps, country-specific policy documents also take it for granted and use equity as a broad principle, without defining it properly and without proposing concrete ways to implement it. In the two countries under study, while UHC policy documents recognise the need to tackle geographic disparities, they actually fail to define binding rules to allocate resources (financial, human, material) in a more equitable way. As for policies aimed at protecting the poorest or the most vulnerable, they are either untargeted (e.g. all children under five or all pregnant women, whatever their socioeconomic status) or face difficulties in identifying the target population (the poorest) [71, 72]. An explanation may be found in the lack of tools that comprehend other types of inequities. Incidentally, “The use of facility data or other administrative sources presents challenges as they […] typically do not collect variables relevant for equity analyses other than geographical location” [4﻿3﻿]. Henceforth, collecting routine data disaggregated on the basis of other stratifiers could be a first step in taking better account of equity in UHC policies, and to better target populations most in need of special care. This would contribute to rendering the UHC policies more effective and more sustainable. In any case, only strong collaboration between policymakers, social scientists, financiers and lawyers can tailor UHC policies to specific country needs, and help translate them into institutional frameworks to facilitate their implementation.

## Data Availability

The documents reviewed are public.

## Abbreviations

ARCH: *Assurance pour le renforcement du capital humain* (Benin)
CBHI: Community-based health insurance
ILO: International Labour Organisation
MoH: Ministry of Health
NHFS: National health financing strategies
NHSP: National health sector plan
PNDS: *Plan national de développement sanitaire* (NHSP)
SDGs: Sustainable Development Goals
UHC: Universal health coverage
WB: World Bank
WHO: World Health Organisation
